# Clinical Notes on Characteristics

**Published:** 1888-03

**Authors:** C. Carleton Smith, Edmund J. Lee

**Affiliations:** Philadelphia; Philadelphia


### CLINICAL NOTES ON CHARACTERISTICS.

C. CARLETON SMITH, M. D., } Philadelphia.

EDMUND J. LEE, M. D., }

### AGARICUS MUSCARIUS.

MENTAL.—The delirium of Agaricus is characterized by its fury, frenzy, and by the strength displayed (resembling Bell., Canth., Hyos., Stram., Tarent., etc.); seems as if intoxicated. Patient is very talkative; constantly changes subjects; runs quickly from one topic to another. (This great loquacity, constantly jumping from subject to subject, is very peculiar to Lachesis. Stramonium has loquacity of a foolish, silly kind, which seems to amuse the patient. With Sticta the patient feels as though she must talk, whether listened to or not. Veratrum, like Stramonium, has much delirium of a religious nature.) The Agaricus patient talks, sings, etc., but does not answer questions. (The patient does not seem inclined to answer or to talk, though he is not ill-humored. Other remedies have this aversion to talking, but they differ from Agaricus. Thus: Obstinate silence, will not answer, China; will not talk, Phos-ac., Rhus, Sulph.; will not answer, Arn., Ars., Hyos.; permits no one to speak to him, Verat.) Generally solicitous about everything, is now quite indifferent. (Great indifference, Nat-m., Phos., Phos-ac., Puls., Sep.; indifferent to those he loves best, yet is interested and converses with strangers, Fluor-ac.) Confusion, cannot find proper word, uses wrong words, worse after exertion, sleepless nights. Indisposed to mental labor.

HEAD.—Vertigo, reeling as if drunken when walking in open air; momentary vertigo from strong sunlight (also Gels., Natr. c.). Protracted mental work or exciting debates cause vertigo. (Also Amm-c., Arn., Borax, Cupr., Gran., Natr-c., Phos-ac., Sep., Sil. Compare with vertigo from reading, etc.) Fainting after moving head, hearing others talk, smelling aromatics, perfumery, or even vinegar. (From various odors, Sang. The Colchicum patient is *very* sensitive to odors; that of fresh eggs nearly causes fainting.) Headache, chiefly a *drawing* pain, which extends into root of nose (Ant-t., Glon., Nux-v.), mornings, sometimes accompanied by epistaxis; dull pain, especially in forehead; must move head to and fro and close the eyes (heaviness, relieved by shaking head, Gels.; pain in temple, relieved by moving head, Cina.); pressing in right side of head, as if a nail had been driven in (Ignatia, etc.); worse when sitting quietly, better when moving slowly about (also Kali iod., Puls., and Sulph.: compare with relief from walking); *headaches of hard drinkers.* (Compare with Acet-acid, Asar., Calad., Nux-v., Op., etc.) Agaricus is especially suited for the headaches of those who have symptoms of chorea, or who become readily delirious in fever or with pain; also headaches with grimaces and twitchings.*

EYES.—Feeling of weariness without having exerted them; sight is dim; flickering before the eyes; range of vision continually changes while reading (also Jabor.); type seems to move (also Amm-c., Cic., Con., Merc., Phys.; on reading by candle-light the lines seem to jump, Bell.); burning in canthi, especially inner; burn and are red, with itching worse from touch; *twitching in eyelids.*

EARS.—Redness, burning, and itching of ears, as if they had been frozen; feels worse from hearing people talk (compare Amm-c., Ars., Con., Mag-m., Marum, Zinc.); every noise causes palpitation. (Natr-p.)

NOSE.—Bleeds in the morning after blowing it (also Brom. and Caust.); epistaxis in old people (also Carb-v.); smell sensitive; vinegar is unbearable (Æth.); dropping of clear water from the nose (Nitr-ac.), without coryza (dropping clear and hot water with coryza is Aconite); offensive and copious nasal discharges; stopped nose, especially when stooping.

FACE.—Lancinating, twitching, or tearing pains; face red and hot or puffy; *twitching of muscles;* paralysis of muscles; one or both corners of mouth drop down and saliva runs out. (Compare with Op., Zinc., etc. Jaws hanging, Ars., Bapt., Lach., Lyc., Mur-ac., Op., Secale; trembling, Op.)

MOUTH, ETC.—Mouth dry; odor very offensive; mercurial apthæ on roof and tongue; bleeding gums; jerks in teeth each time he drinks cold water (throbbing from cold drinks, Carb. an.;

* (NOTE.—In our last issue, we gave under Æthusa the symptom, "thin stools relieve the headache." Dr. J. E. Lilienthal, San Francisco, kindly sends us the following remedies, all having relief of head after stool: Agaricus, Cornus c., Lachnanthes, and Oxal-acid. In addition, we may add Aloe, which has relief after a *complete* stool, and headache when not so. Ptelea has a headache on waking, passing off after stool.)

tearing, Sars., Staph., Sulph.; tearing ameliorated is Bov.) Teeth very sensitive to pressure.

TASTE.—A nauseous sweet; also bitter, offensive, or salty.

TONGUE.—Coated white; tongue tremulous; speech indistinct; soreness, dryness.

THROAT, ETC. — Feeling of dryness, causing contraction; difficult swallowing, with great appetite.

APPETITE, ETC.—Feels better for an hour after eating (Anac.), while he is so exhausted; but great sleepiness remains. Very drowsy after dinner. (Laur., Phos., etc.)

Great hunger, can hardly wait for meals, or much hunger but little appetite.

NAUSEA, ETC.—Eructations tasting of rotten eggs or of apples. Bitter vomit, with stitches in rectum and loin, followed by headache and desire to sneeze. Nausea and vomiting.

STOMACH, ETC.—Cardialgia lasting three hours, after a meal; burning, changing into dull pressure, with nausea. Fullness, pressure, heaviness, etc. From twelve to two P. M., daily, cramp from middle of spine through into stomach; gagging; feeling in legs as if pricked with ice-cold needles.

In loins, a peculiar tensive sensation as if wrenched, less when walking.

ANUS, STOOL, ETC.—Sharp itching in rectum, causes desire to draw upward, but is only relieved by pressing down; biting in after wind passes. *Passes much inodorous flatus.* (Scentless flatus, *Agar.*, Ambr., Arn., Bell., Cann-s., Carb-v., Comoclad., Con. (and cold), Fluor-ac., Hæmat., Lyc., Mang., Marum, Nicc., Nux-j., Phos., Sulph.) Also fetid flatus.

Stool is bilious, grass green; thin, yellow, slimy; pappy, with cutting in abdomen. Diarrhœa mostly in morning after rising; returns after each meal, with rumbling, passage of flatus, and colic. Flatus passed with stool (like Aloe, *Arg-n.*, Gamb., Natr-s., etc.). The pains are before, during, and after stool. Headache worse after stool.

(NOTE.—Stools every morning on rising, etc., is a symptom found under Æth., Calc., Lyc., *Natr-s.*, Nux-v., Oxal-ac., Phos., Psorin., etc. With Aloe and Sulphur the patients are driven out of bed by urging to stool. Cistus and Rhus have this symptom also, but not so characteristically. Oxal-acid has a dark stool at six A. M., with twisting pain in abdomen, and, ike Cistus, has aggravation after coffee. The Petroleum patient is awakened early by urgent desire for a gushing, watery stool with colicky pains.)

URINARY.—Urine clear, watery; apt to be increased in amount (even with diarrhœa). (Copious with diarrhœa is Fluor-ac.) Sensation as if a drop of cold urine passed (also Nitr-acid.). Urine passes at intervals and dribbles away from a cold, shrunken penis. Passes slowly; has to press hard to pass it.

MALE SEXUAL ORGANS.—Great desire, with relaxed penis; after an embrace there is uneasiness, languor, sleepiness (also Sep.; falls asleep during an embrace, Lyc.), pains in limbs, etc., night sweats. (Calc., Natr-m.) Complaints from sexual excesses, old gleets, etc. (See list given after Agnus.)

FEMALE.—*Intolerable bearing down;* cramps as if she would have a child; must lie down. Itching and irritation with strong desire for coition. Menses profuse, with tearing pains in back and abdomen. Headache and toothache before and during menses. Prolapsus after cessation of menses. Nipples itch, burn, and look red. Metastasis of milk to brain.

(NOTE.—This bearing down under Agaricus is very marked, and it is probable that other remedies have been given, in cases where this bearing down was prominent, when Agaricus was the remedy. With Lilium tig. and Natr-m. this bearing down causes the patient to *sit down*. See note under Agnus. The pain in back during menses is found under a number of remedies. It is prominent in cases calling for Kali carb. Under Asarum we find a violent pain in small of back on appearance of menses, which scarcely permits her to breathe.)

LARYNX.—The constriction and oppression make him dread suffocation. Scratching in, after singing.

RESPIRATION.—Difficult, as if chest were too full; *must breathe deeply.* Tension in chest, causing desire to breathe deeply, and sometimes impeding walking. Breathing impeded at night owing to stopped nose. (See under Aconite.) Short breath, making walking difficult.

COUGH.—In isolated attacks ending in sneezing. (Alum., Bad., Bell., Bry., Hepar, Senega.)

HEART.—Sense of oppression in cardiac region. Palpitation, worse in evening (worse lying on left side. Brom., *Cact.*, Dig., Kalm.); violent, strongly felt palpitation with red face; with anxiety and sweat. Burning, shooting pains in region of the heart extending to left shoulder-blade, caused by deep inspiration and worse from coughing, sneezing, etc. (Shooting pain extending to left scapula, Rumex; to left arm, Tarent.) Paralyzed feeling in left arm and hand after palpitation begins. (Numbness in left arm and hand, *Acon.*, Ailan., Kalm.)

BACK.—Sensitive, worse morning. Pain in lumbar and sacral regions during exertion and *while sitting;* pain sore, aching, but not sensitive to touch. Stiffness of neck, back, and between shoulders. Sensation of ants creeping along spine. Numbness and weakness; burning, shooting stitches, etc. Every motion causes pain; pain on stooping; *aching along spine and limbs.* Muscles feel bruised, and on bending forward seem to be too short.

LIMBS, UPPER.—Burning and itching in both hands as if frozen; parts red, hot, and swollen. Trembling and coldness of hands (after writing too much). Drawing, tearing, or laming pains. Fingers stiff from gout.

LOWER.—In the legs, pains (of almost any kind) occur when standing or sitting; more rarely when walking; pains diminish by motion. When standing the pain increases; he is soon obliged to walk or to sit down; weary, tired legs.

Itching, burning, and redness of toes, as if they had been frozen.

SLEEP.—Frequent yawning before spasms or headache; sleep restless from itching and burning of skin; awakes often at night, and is at once wide awake; starts and twitches on falling asleep.

CHILL, FEVER, AND SWEAT.—Very chilly in open air; it strikes through whole body; chilly from slightest movement, or raising bedclothes; heat chiefly on upper part of body, or heat all over, burning; uncovers lower limbs; general heat, with cold finger tips, dry lips, thirst, clean tongue, and pain in limbs; sweat profuse when walking and on slight exertion; greasy, but not offensive; often only on front of body, especially about legs at night; cold on face, neck, and chest.

SKIN.—*Burning, itching, with redness and swelling as if frozen; itching all over* (sometimes comes on after coition), *causing great distress;* without eruption; changing place.

PECULIARITIES.—*Burning and itching of parts as if frozen;* feels as if her limbs did not belong to her; cramps in muscles of limbs, especially when sitting; drawing and *tearing,* especially in limbs, which continue while *sitting* or standing, and go off on motion. The patient feels most comfortable when walking slowly about (Puls.). The pains in lower limbs, especially the knees, almost always come on when sitting or standing, rarely when walking.

Pains in bones forenoon and morning, especially in left tibia (rather better than worse from warmth of bed), better when walking. Slight blows cause ecchymoses. Soreness and bruised feeling after severe epistaxis; after coitus, debility, pains, and weakness in thighs; *twitchings of the muscles* (in low fevers, etc., Hyos.), also of the eyeballs and eyelids; spasmodic motions from simple involuntary motions and jerks of single muscles to a dancing of whole body; *involuntary* movements when awake, *ceasing during sleep.* Symptoms often appear diagonally (right arm, left leg, etc.).

RELATIONSHIP.—Hering gives Agaricus as similar to:

Bell., cerebral excitement, but more in chorea.

Calc., alcoholism, icy-cold feeling on head.

Cann-ind., alcoholism, extravagant fancies.

Cicuta, spasms of eyes; letters go up and down or disappear; objects appear double or black.

Cimic., delirium of alcoholism, chorea, spinal irritation.

Cina, twitching of limbs, chorea; distortions begin with a shriek, extend to tongue, œsophagus, and larynx; continue even through the night.

Codein, eyes twitching on attempting to read; unusual tendency to involuntary twitchings of muscles.

Hyos., typhoid of drunkards; loquacity, dancing, muscular twitchings, and with all tremor, tendency to stupor, and feeble pulse.

Ignatia, hysterical or emotional chorea, sighing, convulsive cough, twitches, laughing and crying.

Jaborandi, spasm of accommodation, vision continually changing; eyes tire easily, are irritable.

Lachesis, loquacious delirium (patient goes rapidly from topic to topic), alcoholism, typhoid of low type, tremulous protrusion of tongue, tremor, feeble pulse, livid extremities.

Natrum ars., vision weakened from condition of health; objects blur when he looks at them for a short time; eyes soon tire and ache; lids disposed to close, cannot keep them open as wide as usual.

Nux vom., chorea, alcoholism, spinal irritation, convulsions, tremor, paraplegic symptoms, enlarged liver.

Opium, alcoholism, chorea, with spasmodic, angular jerks of flexors; hands tremble, slow pulse.

Physost., pain after using eyes, muscæ volitantes, flashes of light, twitching of lids and around eyes.

Pulsat., spinal irritation, chorea, chilblains.

Ruta, itching at inner canthi, eyes burn, ache, feel strained, sight blurred after over use, etc.; spasms of lower lids.

Sepia, icy-cold feeling on head.

Silicea, jerks of head and tongue and limbs. Twitching of limbs during sleep (those of Agar. cease *during* sleep).

Sticta, chorea, with jumping and dancing.

Stram., delirium tremens; singing, laughing, dancing; extravagant recitals; laughs at his own foolish wit; *foolish* loquacity; chorea, with gyratory motions, etc.

Tarent., chorea, one arm and one leg constantly in motion.

Thea, talkative; spinal irritation.

Agaricus is ANTIDOTED by charcoal, coffee, wine, brandy, camphor; is followed well by Bell., Calc., Merc., Op., Puls., Rhus, Silicea.

Acts chiefly upon upper right and lower left sides. Complaints aggravated morning and afternoon. Complaints from sexual excesses.

CONDITIONS (general), aggravation.—On waking; from pressure; on stooping, standing, sitting, or lying; in wet weather; on inspiration; on closing eyes.

AMELIORATION.—When walking, and generally from motion; on opening eyes; in dry weather.

### AGNUS CASTUS.

MENTAL.—Melancholy, listless, dissatisfied, anxious, fear, and weakness. *She is very sad and keeps repeating* that she will die soon. (Guernsey tells us the difference between this fear of death of the Agnus patient and that of the Aconite patient is that the former has no fear of *immediate* death, but thinks it will come soon, after awhile, and that there is no need of doing anything to help her.) *Very absent-minded* (chiefly *Cann-ind.*, Caust., Cham., Graph., Lyc., Nux-m., Phos., Plat., Sep., etc.); reading is difficult, has to read over several times.

HEAD.—Contractive pain above temples from reading (pain in temple from reading, Clem., Mezer., Natr-m., Phys., Sulph.). Tearing pain above eye, with soreness to touch, worse on motion and in evening.

Headache in upper part, as from staying in a room filled with thick, dusky air; looking fixedly at one point relieves (also Sabad.).

Biting itching on scalp (Merc., Puls., Staph.), worse evenings and on falling asleep.

EYES.—Widely dilated pupils; photophobia. Corrosive itching over and on eyebrows and lids and below eyes. (Guernsey adds: This itching is also found on cheeks, chin, tip of nose, perineum, or elsewhere; is relieved by scratching, but soon returns.)

NOSE.—Hard aching in dorsum, as from pressure of a stone; relieved by pressure. Illusions of smell, at one time as of herring, at another like musk.

FACE.—Corrosive itching. Erysipelas on left cheek, spreading from nose over face and head.

MOUTH.—Teeth painful when touched by warm food (Bell., Bry., Calc., Cham., Nux-v., Phos., Puls., Sil.); mouth very dry; saliva tough, drawing out into strings. (Mouth and tongue very dry, saliva like "cotton," *Nux-m.*)

APPETITE, ETC.—Hunger, but food does not agree with him; it makes one feel full and uneasy. Thirstlessness and aversion to drinking.

STOMACH, ETC.—The wind belched smells like old urine allowed to dry on clothes. Nausea felt in epigastrium when standing; later qualmishness in abdomen, with sensation as if intestines were sinking or pressing down; wants to support abdomen with hands.

(NOTE.—Very many remedies have a pressing or bearing down in abdomen; this sensation of Agnus seems to be more a feeling of *weakness* than one of *pressure*. Something like it we find under Staphisagria, which has "a feeling of weakness in abdomen as if it would drop; wants to hold it up." Under Lilium-tig. we find something different: "feeling as if contents were *dragged* down [this feeling extends as far up as chest], wants to support it." There are again remedies having a bearing down as if prolapsus would occur: *Ant-cr.*, *Bell.*, Calc., Con., Cop., LIL-TIG., *Natr-c.*, *Natr-m.*, *Nit-ac.*, Pallad., SEP., Ustil.; must cross limbs to prevent it, *Sepia;* must press upon vulva, *Lil-tig.*, Sepia; must sit down, Lil-tig., Natr-m.).

ABDOMEN.—Sensitive to pressure, or sore to touch. Aching in region of liver; swelling and induration of spleen. Rumbling during sleep.

RECTUM, STOOL, ETC.—Deep fissures at anus; a sore feeling under skin near anus, only when walking. Corrosive itching of the perineum. (Itching of scrotum, *Staph.*) The flatus smells like stale urine after drying on clothes.

Difficult expulsion of soft stools; seemed inclined to re-enter rectum (like Sil.). (Difficult expulsion of soft stool, *Alum.*, Carb-v., China, Phos-ac., Sep., Sil.)

URINARY.—Frequent urination; has to rise twice at night and even once during siesta. Urine dark; disagreeable sensation in back part of urethra after urinating. (Burning in back part after urinating, Kali-b.; feeling as if last drops remained, Arg-n., Kali-b.)

Discharge of prostatic fluid when straining at stool. (Also,

Alum., Amm-c., Anac., Ars., Calc., Cann-i., Carb-v., Caust., Con., Hepar, Ign., Kali-b., Natr-c., Nitr-ac., Phos., Sep., Sil., Staph.; after stool, Caust., Nitr-ac., Phos.)

MALE SEXUAL ORGANS.—*Sexual desire lessened, almost lost; penis small and flaccid* and cold (so that voluptuous fancies cause no erections, etc.). Has usual morning erections but parts are flaccid. After an embrace has an involuntary emission. (Also Acon., Bar., Kali-c., Natr-m., Natr-p., *Phos.*, Rhod.) Testes cold, swollen, hard, and painful.

(Testes cold, Aloe, Berb., Brom., Caps., Merc. Hard or indurated, Aur., Bell., Brom., Clem., Con., Cop., Iod., Merc., Mezer., Nitr-ac., Nux-v., Oxal-ac., Rhod., Spong., Sulph.) Gleet, with want of sexual desire and lack of erections. Induration of testes after suppressed gonorrhœa. (Compare Aur., Bell., Canth., Clem., Natr-c., Nitr-ac., Puls., Rhus, Selen., Sulph., etc.) Pollutions from irritable weakness of parts (Con.). With prostatorrhœa. (See under Urinary Organs.) Gonorrhœa, *with absence of sexual desire and power of erection.*

FEMALE SEXUAL ORGANS.—Hysteria, with maniacal lasciviousness. (Hyos., Bell., etc.) Menses suppressed, with drawing pain in abdomen; sterility with absence of menses and sexual desire.

Transparent leucorrhœa, passes imperceptibly from *very relaxed parts;* stains linen yellow. (Also *Carb-an.*, Chel., *Creos.*, Eupion, Graph., Lach., *Nux-v.*, *Prun.*, Sep., Thuja.) Deficiency of milk in lying-in women (with sadness and the fear of death soon, etc.). (Milk decreased, Dulc., Graph., Puls., Urt-ur.; in rheumatics, Caust.)

RESPIRATORY ORGANS.—Hard pressure upon region of sternum, especially during inspiration. Voice sounds as if it came through wool; has no characteristic sound. (As if mouth were full, Calad.; is toneless, Ars., Plumb., *Stram.*, Sulph.) Cough evening in bed before falling asleep; in paroxysms, with palpitation and nose bleed, mostly in morning.

CHILLINESS, ETC.—Chilly all over, but only hands feel cold to touch. Slight chilliness in evening, followed by heat, with headache. (Natr-m.) No thirst, slight delirium, tormenting and profuse sweat. Flushes of heat, mostly in face, with cold knees, evening in bed (awakens from cold limbs, especially knees, Carb-v.; on waking cold knees, Euphr.; night in bed, *Phos.*)

LIMBS, UPPER.—Sensation of hard pressure in axilla and arm, worse from motion or contact. Swelling of finger joints with arthritic, tearing pains, worse from contact.

LOWER LIMBS.—Lancinating pain in right hip, worse during motion, abating in rest, with debility which obliges sitting down. Sticking in knee, with debility, worse during motion; at rest it is changed to a pressure as if part were luxated. Legs fatigued and swollen toward evening. Feet turn easily when walking on a pavement. (Feet liable to turn when walking, Carb-an., Natr-c., Natr-m., Nux-v., Phos., Sil., Sulph.)

SKIN.—Corrosive itching on different parts of body, obliging one to scratch, which only relieves temporarily. Itching around ulcers in evening.

GENERALITIES.—Constant trembling from internal chilliness, though body is warm to touch. Bruised feeling all over; gouty nodosities. Effects of strains from overlifting. (Also Arn., Borax, *Calc.*, Graph., Lyc., Natr-c., Phos-ac., *Rhus*, Sil., etc.) From sprains of joints. (Also Amm-c., Arn., Bry., *Calc.*, Carb-an., Ign., *Lyc.*, Merc., *Natr-c.*, *Natr-m.*, Nitr., Nitr-ac., Nux-v., Petr., *Phos.*, Puls., Rhus, Ruta, Sulph.) May be indicated in bruises or wounds. Prevents excoriation in walking.

ANTIDOTES TO AGNUS.—Camph., Natr-m. (the headache), strong solution of table salt.

After Agnus follow with Ars., Bry., Ign., Lyc., Puls., Selen., Sulph.

NOTE.—The most marked action of Agnus is the causing of *impotence.* In a hale, hearty man it causes the penis to dwindle, to become cold and flaccid. There is a line of remedies to be compared in this connection. Of Agnus Farrington wrote: "In old men who, having spent their youth and early manhood in practice of excessive venery, are just as excitable in their sexual passions at sixty as at eighteen or twenty, and yet are physically impotent, Agnus is a good remedy."

Argentum, seminal emissions almost every night, without erection, with atrophy of penis. Effects of onanism.

Caladium is indicated after sexual abuse or excesses when there is impotence with mental depression; nightly pollutions without any sexual excitement (without erections, Bell., Cobalt., Con., Gels., Mosch., Sabad., Selen.).

Calcarea is indicated when there is excessive sexual desire (more of a *mental* than physical kind); erections are imperfect during coitus; emissions premature or imperfect, and coitus is followed by vertigo, headache, and weakness in knees (Sep.). (Dioscorea, Agaricus, and Silicea have weakness after coitus.)

China is chiefly useful for the debility following frequent emissions. It has also impotence with sexual desire; is more apt to be needed when these effects follow soon after the excess; Phos-ac. for the more chronic effects.

Cobalt, like Nux vomica, is especially useful for the backache following emissions; it is worse when sitting; (worse sitting, *Agn.*, Berb., Carb-v., *Cob.*, Lyc., Mag-m., Nux-v., Ol-an., Phos., Pic-ac., Puls., Rhus, Sulph., *Tabac.* Zinc.) This drug has also a severe pain in right testicle, better after urinating.

Conium is most useful when extreme melancholy, etc., follows sexual excesses (see Calad.). Emissions without dreams, prostatorrhœa at every emotion. (Without dreams, Cic., *Con.*, Coral., Dig., Guai., Natr-c., *Stann.*)

Digitalis has nightly emissions during sleep, without dreams, with great weakness of sexual organs; with sadness and despair; after emission there is a sensation as of something running out of urethra, and also great weakness.

Lycopodium, like Agnus, the penis is shrunken, cold, and relaxed; there is impotence; erections feeble, and falls asleep during an embrace.

Natr-mur, excessive irritability of sexual instinct, with physical debility. (Weak powers, Agar., Amm-c., Graph., *Ign.*, Men., Nux-m., Nux-v., Selen., Sep., Sil., etc.) Pollutions (even after coitus), followed by weakness, backache, night-sweats, weakness of legs, and melancholy. (Natr-ph. is very similar.)

Nux vomica, sexual desire easily excited, especially in mornings and after too high living. Often easily excited, but power is weak. Emissions while asleep. (Aloe, Alum., Cann-s., Caust., Clem., Coloc., Croton, Cyc., Dig., *Dios.*, Lact., Merc., Natr-c., Ol-an., Phos., Puls., Ran-b., Rhod., Stann., Staph.) There is headache, backache. (Also Cann-i., Cob., Phos., Phos-ac., Staph.) With difficulty in walking. Bad effects of onanism in youths. (After Nux comes Calc., Lyc., Phos-ac., Sulph., etc.)

Phosphorus, impotence following previous over-irritation. Backache as if it would break; burning spots, better from rubbing. Also great sexual excitement, with backache after coitus. (Also Cob., Natr-m., Nux-v., Ph-ac., Selen., Sil., Sulph., Ustil.)

Phos-acid, there is little or no pain, but much debility, after excesses. Emissions are frequent and debilitating, causing melancholy (Calad., Con., Natr-m., Sulph., etc); onanists, who are much distressed by their culpability. (Also Staph.) During coition there is a sudden relaxation of the penis. (Erection too feeble, Agar., Agn., Bar., Calad., Calc., Caust., Hepar, Lach., Lyc., Nux-m., *Phos.*, Sep., Sulph.) Erections without any sexual desire. (Bry., Calad., Eugen., Fluor-ac., Mag-s., Nitr-ac., Nux-v. Spig., Tarent.) The semen is discharged too soon; burning in back and weakness in legs. Scrotum relaxed and hangs down. (Sulph., etc.)

Selenium, weak, debilitated persons, who are too easily fatigued and are overcome by hot weather. After seminal loss there follows headache, irritability, weak back, dribbling of semen during sleep, after stool, or urination. The parts are *relaxed* and hence this dribbling.

Sepia, increased desire, with weakness of genitals (see under Natr-m.). After an emission has burning in urethra, is languid and drowsy (Agar.), weakness in knees (Calc., Dios.).

Silicea, sexual power increased or decreased; weak power, but increased desire; emission too soon. After coition sensation as if paralyzed on right side of head; soreness of limbs, aching all over. All symptoms worse from loss of animal fluids.

Staphisagria, bad effects from onanism, especially when there are dark rings arounds eyes (Zinc.); sallow, sunken face; emaciation; abashed look, backache, weak legs, organs relaxed. After emissions great prostration and dyspnœa.

Sulphur, patient weak, debilitated, with gastric ailments, flushes of heat, cold feet and heat on top of head. Frequent involuntary emissions, exhausting him next morning. Semen is thin, watery; genitals are relaxed; scrotum hangs down; penis cold, erections few and far between; at coitus semen escapes too soon; patient suffers from backache and weakness of limbs; is low-spirited and hypochondriacal. (Selenium is very like Sulphur, but has more relaxation, and Sulphur has offensive sweat on scrotum.)

Ustilago, irresistible tendency to onanism; frequent emissions; is prostrated, dull, has lumbar pains (Cobalt), is despondent, irritable.

Zincum, desire easily excited, but emission too rapid; sometimes it is very difficult; copious prostatorrhœa without any cause; spermatorrhœa without dreams, face pale, sunken, blue rings around. (Staph.)

